# Assessment of Changes in Glycaemic Control and Blood Viscosity Determinants: Does Glycaemia Impact on Haematocrit, Proteinaemia or Dyslipidaemia?

**DOI:** 10.3390/medsci13040303

**Published:** 2025-12-04

**Authors:** Jovita Igwebuike Mbah, Phillip Taderera Bwititi, Prajwal Gyawali, Ezekiel Uba Nwose

**Affiliations:** 1 School of Health and Medical Sciences, University of Southern Queensland, Toowoomba, QLD 4350, Australia; prajwal.gyawali@unisq.edu.au; 2 School of Dentistry and Medical Sciences, Charles Sturt University, Wagga Wagga, NSW 2650, Australia; pbwititi@csu.edu.au

**Keywords:** diabetes mellitus, glycated haemoglobin, haematocrit, lipid profile, serum protein, estimated whole blood viscosity

## Abstract

**Background:** The relationship between glycaemia and the variables of haematocrit, serum total protein and lipids possibly plays a role in pathological processes and hence is a subject of interest. Estimated blood viscosity causes impaired blood flow and is a factor in other vascular diseases. Blood viscosity is correlated with glycated haemoglobin, but the mechanism of this association has not been extensively investigated. **Objective**: To assess if changes in glycated haemoglobin translate into changes in blood viscosity through impact on haematocrit, serum protein or dyslipidaemia. **Method**: This was a clinical laboratory-based retrospective data analysis of patients attending a diabetic clinic. Analysis involved seven variables comprising serum total protein level, high-density lipoprotein cholesterol, total cholesterol, triglyceride, age and glycated haemoglobin. The statistical evaluations were descriptive, comparative and correlational. **Results**: A total of 12,986 sets of data represented the participants in this study. After excluding three with incomplete data of interest, the groups that were created for comparison comprised the following: good glycaemic control (2694), moderate glycaemic control (4075) and poorly controlled (6194). Serum levels of high-density lipoprotein cholesterol, total cholesterol, haematocrit and proteinaemia gradually decreased with worsening glycaemic control, while serum triglyceride and age increased. In the correlation analysis, serum triglyceride level was positively correlated with glycated haemoglobin r = 0.177, while haematocrit and proteinaemia were negatively related, at −0.045 and −0.103, respectively. **Conclusions**: Increase in glycated haemoglobin was inversely related to haematocrit and proteinaemia; therefore, this did not always increase with the determinants of estimated whole blood viscosity. The implication of this is that further studies are required to substantiate the observation of higher whole blood viscosity levels in patients with poorly controlled diabetes.

## 1. Introduction

The viscosity of blood is associated with various diseases, and rheological parameters are often disturbed in diabetes mellitus. Therefore, the detection of microcirculatory disturbances and reversal of rheological impairments are major components of diagnostic and therapeutic strategies in the management of diabetes mellitus [1]. Haematocrit (HCT), together with red blood cell deformability and aggregation, are important determinants of blood viscosity [2]. Previous studies have shown that elevated haematocrit (haemoglobin) is a predictor of T2DM; however, the reasons for this association have not been fully established [3]. Studies have proven that blood viscosity is an independent risk factor for T2DM [4] and increased whole blood viscosity (WBV) is related to impaired peripheral glucose metabolism, T2DM and cardiovascular disease [5]. Glycated haemoglobin (HbA1c), a molecule formed by the attachment of glucose to haemoglobin, is used to measure average glucose levels in the 2–3 months preceding the development of these diseases. Additionally, HCT and blood viscosity are linked and are indicators of cardiovascular risk. Further, HbA1c is widely used as indicator for diabetic control due to its low biological variability and reflection of average glucose concentration over the preceding 8–12 weeks of the onset of the disease [6]. HbA1c monitoring enhances patient compliance with treatment plans and allows adjustments to medications, lifestyle interventions, and management approach based on patients’ glycaemic status [7].

Blood viscosity is reported to be higher in T2DM compared to non-diabetic control subjects [4,6,8]; however, the mechanisms for the elevation in blood viscosity are not clearly understood. Despite this, osmotic diuresis, a consequence of high HbA1c levels, possibly contributes to increased HCT and reduced plasma level resulting in raised viscosity [9]. It has long been reported that blood viscosity of diabetic subjects is about 20% higher than that of normal control subjects [2] and raised plasma cholesterol levels contribute to elevated blood viscosity through the additional effect of hyperglycaemia in patients living with T2DM [10].

Altered levels of haematological parameters have been described in patients with diabetes mellitus [11] and such changes involve red cells, platelets, white cells and coagulation factors [12]. Studies highlight that persistent hyperglycaemia disrupts lipid metabolism, resulting in altered lipid profile [13]. In addition, it has been reported that dyslipidaemia is associated with diabetes mellitus, and patients with high triglycerides and reduced high-density lipoprotein cholesterol (HDL-C) have increased odds of developing diabetes and prediabetes, respectively.

It is known that several variables influence the pathophysiological fluctuations of blood viscosity, including factors such as coagulation and haematological parameters [14]. It is also worth mentioning that diseases or comorbid conditions such as hypertension, metabolic syndrome or renal impairment are known to influence blood rheology [15].

### 1.1. Statement of the Problem and Rationale of Study

HbA1c is reported to be an independent contributor to blood viscosity and HCT. Patients with prediabetes are also reported to have increased blood viscosity and HCT. Elevated HCT and blood viscosity are important components of blood rheology and diabetics have been shown to react typically to HCT variations, suggesting that raised HCT is also associated with increased risk of macrovascular disease [16].

It should be considered that high HbA1c is linked to increased blood viscosity [17,18]. Indeed, blood viscosity is reported to be higher in T2DM than in apparently healthy subjects [4,6,8] and this is linked to HbA1c [5]. It is also known that certain types of anaemia impact on HbA1c [19,20]. Thus, the role of HCT in the elevation of blood viscosity is not clearly understood. Moreover, finding a correlation in a panel test of HCT, serum protein and lipid profile with HbA1c may help to uncover this.

General objective: To assess if changes in HbA1c translate into changes in blood viscosity through impact on HCT or dyslipidaemia.

### 1.2. Specific Objectives

To assess changes in haematology, serum total protein level and lipid profiles with rising glycaemia. Three questions are of interest in this specific objective.

Do HCT levels significantly differ with changes in HbA1c level?

Do serum total protein levels significantly differ with changes in HbA1c level?

Do lipid profile parameters significantly differ with changes in HbA1c level?

The second specific objective is to evaluate correlations between haematology, serum total protein and lipid profile parameters. A fourth question is integral to the second specific objective—viz. How do the HCT, serum total protein levels and lipid profile correlate with HbA1c?

### 1.3. Hypothesis

There are no statistically significant differences in HCT, serum total protein level and lipid profile levels between glycaemic control groups.

There are no statistically significant correlations between HCT, serum total protein or lipid profile levels with glycaemic control groups.

## 2. Materials and Methods

Design: This was a cross-sectional observational quantitative retrospective study. Archived clinical pathology data were collected following a data mining design from the laboratory information system. The retrospective study design meant that data were from multiple time points within a 10-year period, but analysis was performed in a cross-sectional manner. Our evaluation included descriptive, comparative and correlation analyses of the variables of interest.

Setting: Regional New South Wales from a period between 1999 and 2008.

Participants: Patients that attended the diabetic clinic at least twice within the period. That is, the study participants comprise those being monitored for T2DM and dyslipidaemia treatment.

Data: Independent variables collected for this study were limited to age, HbA1c, HCT, serum total protein levels and lipid profile, including total and HDL-C as well as triglyceride levels. The dependent variable was mainly estimated from the whole blood viscosity (eWBV) adopted from [4], which was calculated from HCT and serum total protein using a validated method as in a previous report.

Inclusion criteria: Data were limited to only cases with the seven variables listed above—i.e., age, HbA1c, HCT, serum total proteins, total cholesterol and HDL-cholesterol and triglyceride. The final inclusion criterion was patients who had complete results for all seven variables.

Ethics considerations: Ethical approval was not required as secondary data were collected. No primary data was collected.

Data source: Laboratory Information System of NSW pathology and Private Medical practice in Orange, New South Wales.

Sample size: Number of cases with indices (HCT and serum total protein levels) for eWBV analysis, N = 12,986. After exclusion of data with incomplete variables, the number of cases becomes N = 12,963.

Statistical analysis: Three HbA1c groups were analysed, including Group 1: ≤5.6%, Group 2: 5.7–6.4% and Group 3: ≥6.5%. The mean values of the variables were compared within the three groups of HbA1c controls. Descriptive and comparative evaluation (MANOVA), as well as correlations (Pearson) between haematological and lipid profile parameters as well as serum total protein level and HbA1c group were performed. IBM SPSS statistics version 29 was used as the statistical tool.

## 3. Results

Descriptive statistics of the data show that the mean age of patients was 61 ± 14 years old. The mean levels of all laboratory parameters in the cross-sectional data are normal except in the case of HbA1c, which indicates average poor glycaemic control.(Table 1).

There is high skewness for TG. Kurtosis is within normal levels for all variables except the serum triglyceride level (Table 1).

Further descriptive statistics of variables with stratified HbA1c groups show comparisons of the tested parameters in good control with moderate and poor glycaemic controls (re: Group 1 ≤ 5.6%, Group 2: 5.7–6.4% and Group 3: ≥6.5%). It appears that only age and triglycerides increased with HbA1c, while other variables appear to decrease, as shown in Table 2.

Multivariate analysis shows statistically significant differences between groups for all variables, though with notable limitations in age and haematocrit (Table 3).

Triglyceride was most correlated with HbA1c while HCT and serum total proteins were negatively correlated. However, the levels of correlations were statistically significant but this seems negligible, as shown in Table 4.

## 4. Discussion

The broad objective of this study was to inferentially assess if changes in HbA1c translate into changes in blood viscosity through impact on haematocrit, serum total protein or dyslipidaemia. The presumption is that increased HbA1c is associated with high blood viscosity; this presumption is made using estimated whole blood viscosity (eWBV), a measure of approximate blood viscosity which is a dependent variable underpinned by an increase in independent variables (HCT and/or serum total protein level). Therefore, the inferential investigation is whether an increase in HbA1c is associated with increases in the independent factors of eWBV (HCT and/or serum protein level) as well as lipid profiles.

The observation in Table 1 shows that raised HbA1c is associated with poorly managed diabetes and negative association with serum total cholesterol concentration, possibly due to dyslipidaemia therapy. The increased serum total triglyceride level in this study, though consistent with diabetic dyslipidaemia, implies a possibility of an arbitrarily high value compared to other variables, and, thus, it could also be an outlier. Though HbA1c and HCT are factors of WBV, both do not always move in the same direction. Age correlates positively with HbA1c but negatively with serum total protein concentration and HCT (eWBV indices). This perhaps was due to the confounding effect of age or different types of anaemia; hence, these considerations should be noted when describing the relationship between HbA1c, HCT and eWBV. HbA1c increases with eWBV and eWBV increases with HCT and serum total protein level; therefore, HbA1c should increase with HCT and serum total protein level. The combination of HCT, serum total protein level and HbA1c should therefore increase eWBV.

Demographic data indicates a high SD for age. From the wide SD in age, it can be inferred that a wide range of age groups, younger and older adults, constituted the study data. However, age is a non-modifiable factor, i.e., compared to other variables that are subject to complex physiology. More importantly, elevated triglyceride is an indication that serum triglyceride concentration could be arbitrarily high compared to other variables.

The relevance of this observation is that elevated serum triglyceride levels are consistent with several findings that show an association between high serum levels of triglyceride and glycaemic control [21,22]. This elevated TG level was shown to assist in identifying apparently healthy young men at risk of diabetes, independent of traditional risk factors. There is also a report about elevated plasma triglyceride levels being moderately associated with risk factors for impaired fasting glucose [23].

Further descriptive analyses, as shown in Table 2, based on HbA1c-categorised groups, show the mean level of HbA1c being high in the dataset. Comparing good control with moderate and poor glycaemic control (re: Group 1 ≤ 5.6%, Group 2: 5.7–6.4% and Group 3: ≥6.5%), it was observed that serum total cholesterol, serum HDL-C, serum total protein level and HCT gradually decreased while serum triglycerides and age increased with worsening glycaemia.

The observation of raised HbA1c being associated with diabetes and with poorly controlled cases is supported by the data [24]. Except for triglyceride, all physiologically modifiable variables seem to decrease from good control to poor control of glycaemia. Wide and varied age in the data lends strength to the study. The relevance of this observation is in the implication that efficient management of HbA1c is essential in the control of diabetes. Another observation is the high standard deviation in age across all HbA1c groups. This indicates that a wide range of the population have been captured in the archived pathology database. This is relevant as it represents the age spectrum of individuals with DM (Table 2).

HbA1c is a useful biomarker that reflects the average glucose level over approximately 12 weeks. Thus, it is widely accepted and used as indicator of chronic glycemia [24] and monitoring of HbA1c can be used to assess the effectiveness of treatment and guiding therapeutic interventions [25]. The increasing levels of serum triglycerides and decreasing levels of HDL-C are consistent with the characteristics of diabetic dyslipidaemia, i.e., elevation in serum levels of triglyceride, low-density lipoprotein cholesterol (LDL-C), total cholesterol and decline in HDL-C [26,27]. However, these reports did not indicate the declining level of total cholesterol, hence, the observation from this study requires further elucidation. It is possible that the observations in this study were made due to the effects of medication on the subjects.

It can be observed from Table 3 that for total cholesterol, the degree of significance increases from moderate to poor control while other variables except haematocrit and age show high significance across board. HCT was not significant in the good–moderate group but significant in the good–poor group. Age, on the other hand, was not significant in the moderate–poor group but significant in the moderate–good group. By inference, there should be considerable changes in HCT associated with glycaemia, with slight changes in age greatly impacting the HbA1c and extreme changes in HbA1c being related to changes in HCT. The effect of HbA1c on viscosity may not necessarily be through the impact of HCT; hence, the implication is that the monitoring of these variables will enhance diagnosis and management of diabetes. The relationship between the two variables of HCT and age can be a factor in the diagnosis and management of blood viscosity. Table 3 further showed a lack of significant difference in age between moderate and poor HbA1c groups, and this implies that there is a need to consider adjustment for age in diabetes management strategy.

The current findings are in partial agreement with previous studies that show that individuals with HbA1c-defined prediabetes have elevated blood viscosity and HCT, and that HbA1c is an independent contributor of both blood viscosity and haematocrit [28]. In addition, the combined use of HbA1c and HCT was reported to be better than HbA1c alone as a screening tool for early identification of individuals with gestational diabetes mellitus [29]. However, data from this study showed that HCT and HbA1c do not always move in the same direction as other factors, e.g., different types of anaemia can affect HbA1c value [30].

Some studies have demonstrated that HbA1c is associated with age [31,32], while other studies disagreed [33,34]. Therefore, there are still divergent observations and the present study supports the former. This observation of a positive association of age with HbA1c could be due to comorbidity with other disease conditions, dyslipidaemia medication or a compensatory mechanism to correct anaemia. However, further studies are necessary to explain the influence of age on HbA1c.

The significance to further elucidate the influence of age on glycaemic control hinges on the confounding effects of HCT, which is an independent factor in eWBV determination. That is, age may be a factor in the interpretation of eWBV results of anaemia patients. Regarding correlation analysis (Table 4), which is statistically significant but seemingly negligible, it is observed that HbA1c tends to increase with age, whereas HCT and proteinaemia, which are indices of eWBV, decrease with ageing. A similar significant but weak correlation has been reported previously [14], and it is recognised that the strength of the correlation has merit in the face of statistical significance [35]. Therefore, it can be inferred that age would confound the effect of HbA1c on eWBV. The implication is that adjustment for age is considered in management for patients receiving diabetic therapy. There are divergent and supportive studies around this. First, a study from the research performance site reported that age was negatively correlated with HbA1c [14], albeit on other parameters. On the other hand, the observation presented here is supported by other studies where age was shown to correlate positively with HbA1c [36,37]. However, reports of positive association of HbA1c with whole blood viscosity [38], which disagree with findings of negative correlations with indices of eWBV (HCT and proteinaemia), could be due to the fact that eWBV was calculated from HCT and proteinaemia; although this is adequate for correlating blood viscosity in clinical conditions, it underestimates the impact of red cell deformability and aggregation, which raise viscosity [39]. In other words, eWBV may lack detailed information that could be obtained from a full rheological profile. Whether this could be attributed to the confounding effect of age, triglyceride or other pathophysiological factors constitutes further research.

In terms of hypothesis, Table 3 shows levels of statistical differences while Table 4 show weak but significant correlations. Therefore, the present study highlights the importance of HCT and serum proteins in diabetes management. Both HCT and serum proteins may be inversely correlated with HbA1c (Table 4), and levels are higher in the well-controlled glycaemic group (Table 2). The implication is that eWBV extrapolated from HCT and serum protein is supposed to be higher in people with good glycaemic control, which is not the case. It is recommended that adjustment for other factors such as age becomes necessary in interpreting eWBV results, especially in anaemic patients, and in devising management strategies for diabetic therapy to reduce cardiovascular risk.

Clinical applicability of findings: The clinical applications of this report can be found in its adoption. Although the clinical test has been known and used for at least 30 years [40], the laboratory test is still lacking a pathology service. The first applicability of the findings is the advancement in knowledge among medical scientists about the eWBV method. This applicability is in regards to the interpretation of eWBV results, especially for the formulation of treatment plans in controlling CV risks in diabetic patients.

The novelty of this report is in addressing the assumptions that HbA1c and HCT elevations will lead to an increase in WBV. This report highlights that although an increase in HCT leads to raised WBV, HbA1c and HCT may be inversely related; therefore, the impact on eWBV is confounded, which then requires careful interpretation of results.

Limitations: Samples were restricted to patients with HCT, serum total protein levels, HbA1c and lipid profiles. Low-density lipoprotein cholesterol levels were not assessed because of the sample size being much lower than other variables. Data collection (and hence the results) was from apparently unhealthy individuals who were undergoing laboratory tests for diabetes and dyslipidaemia management.

The presence of comorbidity as well as the therapies and dietary habits of the patients were unavailable from the laboratory information system, and hence were not considered, which could constitute bias. The influence of gender and ethnicity of the patients was also not considered and thus could also be a source of bias, as could the different instruments used in analysing the samples.

Further, this study was focused on T2DM, although T1DM may be incidental. Hence, a lack of insulin level is out of this study’s scope but is acknowledged as a potential limitation. Nevertheless, it is pertinent to note that the comparison between the glycaemic control groups is without bias, as it could be assumed that these unassessed factors’ influence on the assessed parameters should require a separate phenomenon.

## 5. Conclusions

The current cross-sectional observational quantitative retrospective study assessed the following question: “Does glycaemia impact on haematocrit, proteinaemia or dyslipidaemia?” This was with a view to elucidate if changes in HbA1c translate into changes in blood viscosity through their impact on haematocrit, serum protein, or dyslipidaemia. The study has been based strictly on the clinical pathology test results of patients with diabetes and/or dyslipidaemia. Although HbA1c is generally presumed to be a predictor of high blood viscosity and eWBV is dependent on HCT and proteinaemia, the findings from the study indicate that both haematocrit and proteinaemia could be inversely associated with HbA1c. This observation confounds the expectation that blood viscosity would increase as diabetes control worsens. This could be due to confounding factors such as anaemia or other haematological variables that need to be investigated further. The clinical implication of this observation is in interpreting the results of WBV and in formulating a treatment strategy in diabetic patients to control cardiovascular risk. Care should also be taken in the management of dyslipidaemia in diabetes due the observation of lipids in this study.

## Figures and Tables

**Table 1:** Descriptive statistics of cross-sectional data.

Variable	N *	Mean	SD	Skewness	Kurt	Minimum	Maximum
Age (years)	12,986	61.35	13.78	−0.51	0.12	8.00	97.00
Total cholesterol (mmol/L)	12,985	4.94	1.10	0.74	3.19	1.30	16.60
Triglyceride (mmol/L)	12,968	1.96	1.61	9.75	230.07	0.17	62.09
HDL-C (mmol/L)	12,986	1.22	0.38	1.05	2.02	0.08	3.70
HbA1c %	12,986	6.78	1.61	1.52	3.49	2.40	18.60
Serum protein (g/dL)	12,982	72.11	5.20	0.04	0.99	44.00	109.00
Haematocrit %	12,986	0.43	0.04	−0.43	1.11	0.18	0.58

* Among these data, the valid N (listwise), i.e., complete with all variables, is N = 12,963.

**Table 2:** Descriptive statistics of variables with glycaemic groups.

Variables	Group	Mean	Std. Deviation	N
Total Cholesterol (mmol/L)	Good control	5.0765	1.04695	2694
	Moderate control	5.0113	1.08434	4075
	Poor control	4.8395	1.12299	6194
Serum Triglyceride (mmol/L)	Good control	1.6959	1.20017	2694
	Moderate control	1.8378	1.30699	4075
	Poor control	2.1506	1.86713	6194
Serum HDL-C (mmol/L)	Good control	1.2960	0.39682	2694
	Moderate control	1.2581	0.38399	4075
	Poor control	1.1566	0.35245	6194
HbA1c (%)	Good control	5.1361	0.37934	2694
	Moderate control	5.9853	0.25307	4075
	Poor control	8.0084	1.49391	6194
Serum Protein (g/L)	Good control	72.8615	5.10591	2694
	Moderate control	72.3801	5.20583	4075
	Poor control	71.6015	5.19413	6194
Haematocrit (%)	Good control	0.4321	0.03733	2694
	Moderate control	0.4318	0.03907	4075
	Poor control	0.4267	0.04217	6194
Age (years)	Good control	55.1778	15.03154	2694
	Moderate control	62.7448	13.10084	4075
	Poor control	63.1182	12.84780	6194

**Table 3:** MANOVA outcomes in comparison of HbA1c groups.

Independent Variable			Sig.	95% Confidence Interval	
				Lower	Upper
Total cholesterol (mmol/L)	Good control	Moderate control	0.017	0.012	0.118
		Poor control	0.000	0.187	0.287
	Moderate control	Good control	0.017	−0.118	−0.012
		Poor control	0.000	0.129	0.215
	Poor control	Good control	0.000	−0.287	−0.187
Serum triglyceride (mmol/L)	Good control	Moderate control	0.000	−0.215	−0.129
		Moderate control	0.000	−0.219	−0.065
		Poor control	0.000	−0.526	−0.383
	Moderate control	Good control	0.000	0.065	0.219
		Poor control	0.000	−0.375	−0.250
	Poor control	Good control	0.000	0.383	0.526
		Moderate control	0.000	0.250	0.375
Serum HDL-C (mmol/L)	Good control	Moderate control	0.000	0.020	0.056
		Poor control	0.000	0.123	0.156
	Moderate control	Good control	0.000	−0.056	−0.020
		Poor control	0.000	0.087	0.116
	Poor control	Good control	0.000	−0.156	−0.123
		Moderate control	0.000	−0.116	−0.087
Serum protein (g/L)	Good control	Moderate control	0.000	0.229	0.734
		Poor control	0.000	1.026	1.494
	Moderate control	Good control	0.000	−0.734	−0.229
		Poor control	0.000	0.574	0.983
	Poor control	Good control	0.000	−1.494	−1.026
		Moderate control	0.000	−0.983	−0.574
Haematocrit (%)	Good control	Moderate control	0.722	−0.002	0.002
		Poor control	0.000	0.004	0.007
	Moderate control	Good control	0.722	−0.002	0.002
		Poor control	0.000	0.003	0.007
	Poor control	Good control	0.000	−0.007	−0.004
		Moderate control	0.000	−0.007	−0.003
Age (years)	Good control	Moderate control	0.000	−8.220	−6.914
		Poor control	0.000	−8.547	−7.334
	Moderate control	Good control	0.000	6.914	8.220
		Poor control	0.167	−0.904	0.157
	Poor control	Good control	0.000	7.334	8.547
		Moderate control	0.167	−0.157	0.904

**Table 4:** Results of correlation analysis of variables.

	Age	TCHOL	TRIG	HDL-C	HBA1C	PROT	HCT
Age	1						
TCHOL	−0.114	1					
TRIG	−0.04	0.305	1				
HDL-C	0.013	0.264	−0.264	1			
HBA1C	0.048	−0.006	0.177	−0.143	1		
PROT	−0.105	0.153	0.064	0.086	−0.103	1	
HCT	−0.151	0.112	0.047	−0.045	−0.009	0.203	1

## Data Availability

The original contributions presented in this study are included in the article. Further inquiries can be directed to the corresponding authors.
